# Dispatch Decisions and Emergency Medical Services Response in the Prehospital Care of Status Epilepticus

**DOI:** 10.5811/westjem.21266

**Published:** 2025-05-18

**Authors:** Robert P. McInnis, Andrew J. Wood, Courtney L. Shay, Anna A. Haggart, Remle P. Crowe, Elan L. Guterman

**Affiliations:** * University of California, San Francisco, Department of Neurology, San Francisco, California; † Weill Cornell Medical College, Department of Neurology, New York, New York; ‡ University of California, San Francisco, Department of Emergency Medicine, San Francisco, California; § University of Nebraska Medical Center, Omaha, Nebraska; ¶ University of California, San Francisco, Philip R. Lee Institute for Health Policy Studies, San Francisco, California; || ESO Solutions, Inc, Austin, Texas; # University of California, San Francisco, Department of Neurology, Weill Institute for Neurosciences, San Francisco, California

## Abstract

**Objective:**

Emergency medical dispatch is intended to ensure that emergency medical services (EMS) allocate appropriate resources for the treatment of patients with status epilepticus (SE). However, it is unclear whether dispatch algorithms accurately identify those patients having a seizure-related medical emergency and how dispatch algorithms influence what prehospital resources are allocated for the encounter.

**Methods:**

We performed a cross-sectional analysis of prehospital encounters for SE using data from the 2019 ESO Data Collaborative. We included patients who were ≥18 years of age, had an EMS diagnostic impression of SE, and did not have a cardiac arrest. We examined the dispatch-determined complaint designated by the emergency medical dispatch (EMD) code, dispatch-determined level of acuity (A, B, C, D), ambulance response, and training level of the responding prehospital professional.

**Results:**

Of the 18,515 patient encounters for SE with an EMD code, 8,279 (44.9%) were women, and the mean age was 40.0 years (SD 19.7). There were 13,829 (75%) encounters that received a dispatch code for seizures/convulsions and 4,686 (25%) with a dispatch code for a non-seizure-related condition. Among encounters for SE identified by dispatch as seizures/convulsions, 6,412 (46.4%) were designated high acuity, 6,626 (63.6%) were designated low acuity, and the majority received emergent ambulance responses (98.1% among those designated high acuity and 81.8% among those designated low acuity) and an Advanced Life Support-trained responder (93.7% among those designated high acuity and 92.7% among those designated low acuity). Median response times were similar for all acuity levels (9.1, 8.8, 9.1, and 8.3 minutes for A-D, respectively).

**Conclusion:**

Approximately one-fourth of SE cases were categorized as a non-seizure related condition at dispatch, and fewer than half received the highest acuity determinant code. Despite this, dispatch-assigned acuity did not have a strong relationship with the ambulance response or training level of the EMS responder or response time, suggesting that use of dispatch algorithms might be further optimized and highlighting a potential area to improve quality of EMS care.

## INTRODUCTION

Status epilepticus (SE) is a neurologic emergency that relies on emergency medical services (EMS) for treatment outside the hospital setting, with high-quality evidence demonstrating that rapid EMS stabilization and treatment with a benzodiazepine improves SE outcome.^1^ Prehospital data suggest that seizures prompt a substantial portion of calls to EMS in both pediatric and adult populations (≈6–8%) and that SE represents 6% of seizure-related emergency department visits, highlighting the magnitude of this problem.^2^,^3^ Emergency medical dispatch (EMD) is the system responsible for fielding 9-1-1 requests and allocating resources using a preset algorithm. Thus, EMD serves a critical role in ensuring that patients with SE receive rapid, effective treatment.^4^

In an optimally functioning EMD system, high-acuity medical scenarios would be accurately identified and rapidly receive ambulance care delivered by personnel trained in Advanced Life Support (ALS) capable of providing an appropriate level of treatment (eg, delivery of certain medications, intubation for airway or breathing compromise). Conversely, low-acuity responses would receive less rapid ambulance care delivered by personnel with Basic Life Support (BLS) rather than ALS training.^5^–^11^ However, there are likely large gaps in the ability to distinguish between low-acuity^12^,^13^ and high-acuity scenarios.^14^,^15^ The performance of EMD systems for patients with SE in particular is unclear. We, therefore, aimed to describe dispatch-related decisions for patients with SE using a national prehospital cohort. Our analysis focused on agencies that used the Medical Priority Dispatch System (MPDS) for assigning EMD codes. The MPDS is one of the most common systems used in the United States and internationally, whereby dispatch telecommunicators follow a rigid, predetermined algorithm to code prehospital encounters with a specific complaint (eg, seizure) and acuity level. Variability exists between EMS agencies with respect to how resources are allocated for determinant codes, although these are preset and not up to the telecommunicator to decide at the time of a 9-1-1 call.

## METHODS

### Study Design and Data Source

This was a cross-sectional analysis of prehospital encounters for patients with SE using data from the ESO Data Collaborative from January 1–December 31, 2019. The collaborative, which compiles data from more than 3,000 EMS agencies, fire departments and hospitals, is the research arm of ESO Solutions, Inc (Austin, TX), a company that provides electronic medical record software to EMS agencies across the US. Deidentified data elements are available for research purposes. These include dispatch codes and clinical details for each prehospital encounter, which are compliant with National EMS Information System version 3. We used this data to examine dispatch decisions for patients with SE, determining whether patients were categorized as medical emergencies, the priority of the ambulance response, and the training level of the responding prehospital professional. Our primary outcome was ambulance response time. Our study protocol was approved by the Institutional Review Board of the University of California, San Francisco.

**Population Health Research Capsule**

What do we already know about this issue?

*Emergency medical dispatch (EMD) is critical for allocating resources for status epilepticus (SE), but its accuracy and impact on prehospital care are unclear.*

What was the research question?

*Does EMD classify SE as high acuity, and how does this influence ambulance response time and receipt of ALS-trained responders?*

What was the major finding of the study?

*Less than 50% of SE cases were designated high acuity. Median ambulance response times fell within 2 minutes of each other, and most cases received ALS responders (> 90% for both high and low acuity).*

How does this improve population health?

*Findings demonstrate gaps in dispatch algorithms and a path for improving EMS care for SE, potentially leading to better outcomes.*

### Study Population

Inclusion criteria included encounters for patients who were ≥18 years of age, evaluated for a 9-1-1 call, had an EMS primary or secondary primary diagnostic impression of SE, and received an EMD code that adhered to the MPDS. (We excluded agencies that used alternative EMD systems).^16^ An EMS diagnostic impression for SE has been demonstrated to have high specificity and moderate sensitivity for identifying generalized convulsive SE; restricting encounters with an EMS diagnostic impression for SE to those that receive benzodiazepine results in only modest improvement to specificity, with a large reduction in sensitivity.^17^ Emergency responders select impressions from a set of options that can vary across EMS agencies, which include seizure- or epilepsy-related impressions that indicate the presence or absence of SE (eg, “Seizure,” “Seizure without SE,” “Seizure with SE,” “Epilepsy with SE,” etc). These impressions did not specifically indicate whether convulsive activity had stopped, since SE may manifest with non-convulsive activity/persistent altered mental status. We excluded from our analysis EMS agencies that did not use the MPDS, since these encounters were not recorded in the ESO database. Patients who received cardiopulmonary resuscitation were excluded given the assumption that cardiac arrest would be the main complaint identified by dispatch. We also excluded patients with no EMD code.

### Measurements

We used the MPDS EMD code to determine the complaint and acuity level identified for each encounter. The MPDS EMD codes include a protocol number and letter (A [Alpha], B [Bravo], C [Charlie], or D [Delta]). Protocol number 12 designates seizures/convulsions. The letter specifies the acuity level in quasi-escalating priority (A = low priority, B = middle priority, C = possibly life-threatening, and D = life-threatening), with examples as follows:

- 12A: patient with seizures that have terminated, and breathing verified
- 12B: patient with seizures that have terminated but breathing not verified
- 12C: patient with seizures and a high-risk comorbidity (diabetes, heart disease, or pregnancy)
- 12D: patient with seizures who is not breathing, or ongoing seizure activity

We created a binary indicator of whether the EMD protocol number was 12, specifying the complaint as seizure/convulsion. We defined acuity level as a categorical variable (A, B, C, or D) and created a binary indicator of whether acuity level was low (A, B, or C) or high acuity (D). Patient characteristics included age, sex, race, and Hispanic ethnicity.

Characteristics of the prehospital response included whether the ambulance response was categorized as emergent vs non-emergent, whether the responders had ALS or BLS training, ambulance response time, total prehospital encounter time, whether the encounter occurred in a rural area (according to the US Census Bureau classification), and the EMS agency staffing structure and EMS agency governing structure. Advanced Life Support included ambulances with responders capable of providing specialty critical care services. Response time was defined as the number of minutes between dispatch receiving a 9-1-1 call and the ambulance arriving on scene. Staffing structure was categorized as an agency having paid personnel, volunteers, or a mix. Governing structure was categorized as agency overseen by a non-profit/community organization, hospital, fire department, governmental body, or a private entity.

### Statistical Analysis

We examined patient and agency characteristics of all included encounters, comparing encounters that dispatch identified as seizures/convulsions vs not and among encounters identified as seizures/convulsions, comparing encounters that dispatch identified as high acuity vs low acuity.

For encounters with EMD code for seizures/convulsions we examined the relationship between the acuity assigned by dispatch and agency response in two ways. First, we examined differences in the proportion of encounters that had 1) ambulance response categorized as emergent vs non-emergent and 2) ALS- vs BLS-trained team of responders among encounters identified as high acuity vs low acuity. Second, we examined differences in the speed of the response by calculating the 25^th^, 50^th^, and 75^th^ percentile response time and total prehospital encounter time for encounters within each acuity level (A, B, C, and D). We examined the adjusted difference in response time and total prehospital encounter time. Adjustment was derived by fitting multilevel mixed-effects linear regression models with agency as a random effect and with patient age and sex as fixed effects. We stratified these estimates by agency rurality to account for the longer distances required for travel in rural areas. We also compared the rate of emergent vs emergent and ALS vs BLS assignment among encounters with non-seizure/convulsion EMD vs non-seizure EMD codes.

We performed two sensitivity analyses. To address the fact that some encounters have multiple ambulances dispatched to responder care, we repeated our analyses only including encounters linked to the first responding ambulance. To increase the specificity of our definition for SE, we repeated our analyses including only encounters for patients who were diagnosed with SE and received a benzodiazepine.^17^

## RESULTS

**Figure 1:**
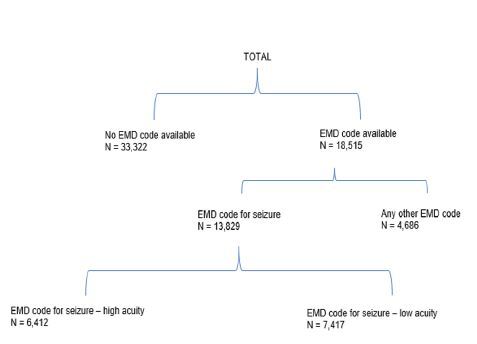
Number of patients with and without EMD codes in a study of dispatch acuity for status epilepticus calls. *EMD*, emergency medical dispatch.

There were 18,515 prehospital encounters for patients with SE that met the inclusion criteria (Figure 1). Patients had a mean age of 40.0 years (SD 19.7), and 44.9% were identified as women. Most encounters occurred in urban locations and involved non-volunteer agencies **(**Table 1). Characteristics of the encounters excluded from our main analysis that had no EMD code are described in Appendix Table 1.

Among all encounters with an EMD code, 13,829 (75%) received a code for seizures/convulsions and 4,686 (25%) received a code for non-seizure-related conditions, of which 8.8% comprised a category of “other,” which included a long list of labels that were not easily included in any existing category (Table 1, Appendix Table 2). Across the 13,829 encounters with an EMD code for seizures/convulsions, 2,651 (19.2 %) were assigned acuity level A, 1,059 (7.7%) acuity level B; 2,916 (21.1%) acuity level C; and 6,412 (46.4%) acuity level D. Compared to low- acuity encounters, encounters assigned a high acuity (acuity level D) had patients with lower Glasgow Coma Scale scores and no clinically meaningful differences in the initial heart rate, blood pressure or oxygen saturation (Appendix Table 3).

There were 6,291 (98.1%) encounters with an EMD for seizure assigned a high acuity that received an emergent ambulance response and 5,855 (91.3%) that received an ALS-trained prehospital responder. There were 5,421 (81.8%) encounters with EMD for seizure assigned a low acuity that received emergent ambulance response and 6,101 (92.1%) that received an ALS-trained prehospital responder. The rate of emergency response and ALS assignment for encounters with an EMD code for seizure/convulsion compared to encounters with a non-seizure EMD code was similar (Appendix Table 4). Median response times across encounters’ associated acuity level fell within two minutes of each other (9.1, 8.8, 9.1 and 8.3 minutes for level A, B, C, and D, respectively). After adjustment, we found that the response times for 12B, C and D were faster than those for 12A (Table 2). Sensitivity analyses restricting to encounters involving the first responding unit and encounters involving benzodiazepine administration did not change the results (Appendix: Tables 5, 6, and 7). The rate of receipt of a benzodiazepine was 15.2% for 12A encounters, 12.8% for 12B, 15.4% for 12C, and 25.9% for 12D. Within each category, midazolam was the benzodiazepine administered in >90% of encounters.

## DISCUSSION

We found that 25% of patients with SE are not categorized as having a seizure by EMD and fewer than 50% receive the highest acuity determinant code. However, the ambulance response and training level of the responding prehospital professionals did not differ meaningfully between encounters identified as low vs high acuity.

Our finding that telecommunicator mis-categorization occurs frequently is consistent with the literature on other medical emergencies, including cardiac arrest^18^,^19^ and stroke.^20^–^23^ The extent to which this is explained by our reliance on bystanders to provide information or disease mimics is unclear. Our findings also suggest that dispatch codes have limited ability to distinguish acuity, but this does not substantially influence downstream EMS care for SE. Similar to prior work from a single city demonstrating that acuity level correlated poorly with benzodiazepine receipt for patients with seizure,^24^ our analysis of a large EMS cohort found that the rate of benzodiazepine administration was ≤25% for all patients with SE regardless of dispatcher-assigned acuity level, consistent with literature demonstrating low rates of prehospital SE treatment overall.^25^

Although our findings provide reassurance that high-acuity resources are deployed consistently for a true emergency like SE, they may reflect a well-recognized tendency for prehospital dispatch systems to over-triage,^15^,^26^ or in part may be explained by aspects of dispatch algorithms mediated at a local/agency level that could not be characterized in this analysis, representing a worthy focus of future investigation.

## LIMITATIONS

This analysis was limited to agencies that use ESO and MPDS for prehospital medical documentation, limiting generalizability. We also did not account for MPDS versions used by various agencies or the policies employed at a local/agency level; both factors may dictate what resources are allocated for each determinant code and, thus, are likely to influence dispatch patterns. Dichotomization of the acuity-level variable in our analysis facilitated interpretation of trends in this population at the expense of capturing more nuanced information about why lower acuity determinant codes may trigger utilization of an emergent priority of ALS response. We were unable to explore these complexities using our analysis, which is a limitation and worthy topic of future investigation.

We relied on EMS diagnostic impressions to identify SE but were unable to confirm whether the patients were truly experiencing SE at the time when the dispatch telecommunicator was responding to the 9-1-1 call. An EMS diagnostic impression for SE has only moderate sensitivity; therefore, we may have excluded some patients with true SE in our cohort from this analysis. We excluded encounters with no EMD code but were unable to verify the cause for these missing data, which may have included lack of data entry at an agency level, or that the dispatcher did not enter an EMD code for a specific encounter. For encounters with an ALS response, we were unable to verify whether a BLS response would have been sent if available. Similarly, we lacked information on the proportion of responders at each EMS agency who had ALS training and, thus, we were unable to determine whether the high proportion of encounters with an ALS-trained responder was merely reflective of agency staffing.

## CONCLUSION

Our findings demonstrate that application of the current emergency medical dispatch system for patients with status epilepticus may not be optimized but does not lead to substandard allocation of resources. More investigation is needed to determine how dispatch algorithms can be better designed to improve the quality of prehospital care.

## Supplementary Information















## Figures and Tables

**Table 1:** Characteristics of prehospital encounters with status epilepticus with an emergency medical dispatch code (EMD) for seizure, or any other EMD code.

	EMD code for convulsions/seizures N = 13,829	Any other EMD code N = 4,686	Seizure code, high-acuity level N = 6,412	Seizure code, low-acuity level N = 7,417
**Patient**				
Age, mean (SD)	38.8 (19.1)	43.6 (20.9)	40.8 (19.0)	37.0 (18.9)
Female gender y/n	6,095 (44.2%)	2,184 (46.8%)	2,873 (44.9%)	3,222 (43.6%)
Race				
White	6,787 (49.1%)	2,738 (58.4%)	2,990 (46.6%)	3,797 (51.2%)
Black	5,749 (41.6%)	1,423 (30.4%)	2,806 (43.8%)	2,943 (39.7%)
Asian	71 (0.5%)	31 (0.7%)	25 (0.4%)	46 (0.6%)
Other	693 (5.0%)	238 (5.1%)	332 (5.2%)	361 (4.9%)
Unknown	529 (3.8%)	256 (5.5%)	259 (4.0%)	270 (3.6%)
Hispanic ethnicity y/n	939 (6.8%)	311 (6.6%)	457 (7.1%)	482 (6.5%)
Rural	387 (2.8%)	169 (3.6%)	158 (2.5%)	229 (3.1%)
Region				
Northeast	99 (0.7%)	47 (1.0%)	9 (0.1%)	90 (1.2%)
Midwest	2,568 (18.6%)	808 (17.3%)	1,128 (17.6%)	1,440 (19.4%)
South	9,256 (67.0%)	2,752 (58.8%)	4.510 (70.4%)	4,746 (64.1%)
West	1,891 (13.7%)	1,075 (23.0%)	758 (11.8%)	1,133 (15.3%)
**Agency**				
Status				
Mixed	1,914 (13.8%)	560 (12.0%)	858 (13.4%)	1,056 (14.2%)
Non-volunteer	11,916 (86.2%)	4,125 (88.0%)	5,554 (86.6%)	6,360 (85.7%)
Volunteer	1 (<1%)	1 (<1%)	0 (0.0%)	1 (<1%)
Type				
Community, non-profit	9,699 (70.1%)	2,922 (62.4%)	4,632 (72.2%)	5,067 (68.3%)
Fire department	1,014 (7.3%)	399 (8.5%)	443 (6.9%)	571 (7.7%)
Governmental, non-fire	2,623 (19.0%)	1,143 (24.4%)	1,135 (17.7%)	1,488 (20.1%)
Hospital	31 (0.2%)	13 (0.3%)	0 (0.0%)	31 (0.4%)
Private, non-hospital	462 (3.3%)	209 (4.5%)	202 (3.2%)	260 (3.5%)

*EMD*, emergency medical dispatch.

**Table 2:** Prehospital encounters for EMS: response times*, and adjusted† differences in response times, stratified by emergency medical dispatch code acuity level (A, B, C, and D, with each successive letter implying increasing acuity), emergency designation, and service level of the unit dispatched to the scene.

	Response time (minutes)			Adjusted difference in response time (minutes)	
	
	25th percentile	Median	75th percentile	Coefficient	95% CI
**EMD Code**					
12A	6.6	9.1	12.9	Ref	
12B	6.6	8.8	11.7	−1.0	−1.3, −0.7
12C	6.8	9.1	12.0	−0.9	−1.1, −0.6
12D	6.2	8.3	11.1	−1.7	−1.9, −1.5
12-NOS	5.5	7.7	11.0	−2.0	−2.7, −1.3
**Priority**					
Not emergency	7.3	10.2	13.8	Ref	
Emergency	6.3	8.5	11.4	−2.0	−2.2, −1.7
**Service Level of EMS Unit**					
BLS	6.9	8.6	10.9	Ref	
ALS + Specialty Critical Care	6.4	8.6	11.7	−0.14	−0.6, 0.3

* Defined as the number of minutes between dispatch receiving an emergency call and the ambulance arriving on scene.

† Adjusted differences in response times were derived by fitting multilevel mixed-effects linear regression models with agency as a random effect, to estimate the difference in response time between acuity, priority, and service levels, adjusting for patient age and sex.

*EMS*, emergency medical services; *CI*, confidence interval; *EMD*, emergency medical dispatch; *NOS*, Not otherwise specified; *BLS*, Basic Life Support; *ALS*, Advanced Life Support.
