# Supplementary material for: Dispatch Decisions and Emergency Medical Services Response in the Prehospital Care of Status Epilepticus
Source: West J Emerg Med. 2025 May 18;26(3):549–55. doi: 10.5811/westjem.21266 (PMC12208027; doi:10.5811/westjem.21266)
Supplement: Supplementary file 1 [file wjem-26-549-s001.docx]

**Table 1 (Appendix)**. Characteristics of prehospital encounters with and without emergency medical

dispatch (EMD) codes

|  | **All encounters** | **EMD code available** | **No EMD code** |
| --- | --- | --- | --- |
|  | N = 51837 | N = 18515 | N = 33322 |
| **Patient** |  |  |  |
| Age, mean (SD) | 39.6 (19.9) | 40.0 (19.7) | 39.4 (20.0) |
| Female gender y/n | 23705 (46.0%) | 8279 (44.9%) | 15426 (46.7%) |
| Race |  |  |  |
| White | 31125 (60.0%) | 9525 (51.4%) | 21600 (64.8%) |
| Black | 15553 (30.0%) | 7172 (38.7%) | 8381 (25.2%) |
| Asian | 272 (0.5%) | 102 (0.6%) | 170 (0.5%) |
| Other | 2861 (5.5%) | 931 (5.0%) | 1930 (5.8%) |
| Unknown | 2026 (3.9%) | 785 (4.2%) | 1241 (3.7%) |
| Hispanic ethnicity y/n | 4327 (8.3%) | 1250 (6.8%) | 3077 (9.2%) |
| Rural | 3203 (6.2%) | 556 (3.0%) | 2647 (7.9%) |
| Region |  |  |  |
| Northeast | 1559 (3.0%) | 146 (0.8%) | 1413 (4.3%) |
| Midwest | 11131 (21.5%) | 3376 (18.3%) | 7755 (23.4%) |
| South | 34438 (66.7%) | 12008 (64.9%) | 22430 (67.6%) |
| West | 4530 (8.8%) | 2966 (16.0%) | 1564 (4.7%) |
| **Agency** |  |  |  |
| Status |  |  |  |
| Mixed | 9456 (18.3%) | 2474 (13.4%) | 6982 (21.0%) |
| Non-volunteer | 41960 (81.0%) | 16039 (86.6%) | 25921 (77.9%) |
| Volunteer | 383 (0.7%) | 2 (<1%) | 381 (1.1%) |
| Type |  |  |  |
| Community, non-profit | 33357 (64.3%) | 12621 (68.2%) | 20736 (62.2%) |
| Fire department | 7403 (14.3%) | 1413 (7.6%) | 5990 (18.0%) |
| Governmental, non-fire | 7677 (14.8%) | 3766 (20.3%) | 3911 (11.7%) |
| Hospital | 137 (0.3%) | 44 (0.2%) | 93 (0.3%) |
| Private, non-hospital | 3263 (6.3%) | 671 (3.6%) | 2592 (7.8%) |
