# Supplementary material for: Dispatch Decisions and Emergency Medical Services Response in the Prehospital Care of Status Epilepticus
Source: West J Emerg Med. 2025 May 18;26(3):549–55. doi: 10.5811/westjem.21266 (PMC12208027; doi:10.5811/westjem.21266)
Supplement: Supplementary file 2 [file wjem-26-549-s002.docx]

**Table 2 (Appendix).** Distribution of types of emergency medical dispatch codes (EMD) associated with an emergency medical services diagnosis of status epilepticus

| **EMD code** | **Diagnosis** | **N (%)** |
| --- | --- | --- |
| **1** | Abdominal pain | 6 (<0.1%) |
| **2** | Allergies/Envenomations | 5 (<0.1%) |
| **5** | Back Pain (non traumatic) | 2 (<0.1%) |
| **6** | Breathing Problems | 139 (0.8%) |
| **7** | Burns | 2 (<0.1%) |
| **8** | Carbon Monoxide | 2 (<0.1%) |
| **9** | Cardiac/Resp Arrest/Death | 135 (0.7%) |
| **10** | Chest pain (non traumatic) | 85 (0.5%) |
| **11** | Choking | 30 (0.2%) |
| **12** | Seizures | 13829 (74.7%) |
| **13** | Diabetic problems | 43 (0.2%) |
| **14** | Drowning | 878 (<0.1%) |
| **15** | Electrocution | 23 (<0.1%) |
| **16** | Eye problems | 133 (0.7%) |
| **17** | Falls | 196 (1.1%) |
| **18** | Headache | 15 (0.1%) |
| **19** | Heart problems | 40 (0.2%) |
| **20** | Heat/Cold | 34 (0.2%) |
| **21** | Hemorrhage/Laceration | 49 (0.3%) |
| **23** | Overdose/Poisoning | 46 (0.3%) |
| **24** | Pregnancy/childbirth | 2 (0.01%) |
| **25** | Pyschiatric | 28 (0.2%) |
| **26** | Sick Person | 280 (1.5%) |
| **27** | Stab/Gunshot/Penetrating Trauma | 2 (<0.1%) |
| **28** | Stroke/TIA | 202 (1.1%) |
| **29** | Traffic Injuries | 67 (0.4%) |
| **30** | Traumatic injuries | 21 (0.1%) |
| **31** | Unconscious/Fainting | 913 (4.9%) |
| **32** | Unknown problems (person down) | 159 (0.9%) |
| **33** | Transfer/Interfacility | 269 (1.5%) |
| **36** | Pandemic/Epidemic/Outbreak | 1 (<0.1%) |
| **37** | Interfacility transfer | 143 (0.8%) |
| **38** | Advanced send | 6 (<0.1%) |
| **Other** |  | 1629 (8.8%) |
