# Supplementary material for: Dispatch Decisions and Emergency Medical Services Response in the Prehospital Care of Status Epilepticus
Source: West J Emerg Med. 2025 May 18;26(3):549–55. doi: 10.5811/westjem.21266 (PMC12208027; doi:10.5811/westjem.21266)
Supplement: Supplementary file 3 [file wjem-26-549-s003.docx]

| **Table 3 (Appendix).** Baseline clinical characteristics of prehospital encounters for status epilepticus | | | | | | | | | |
| --- | --- | --- | --- | --- | --- | --- | --- | --- | --- |
|  | **Low Acuity** | | | | | **High Acuity** | **Acuity not specified** | p^b^ | p^c^ |
|  | Overall | 12A | 12B | 12C | p^a^ | 12D |  |  |  |
|  | **6626** | **2651** | **1059** | **2916** |  | **6412** | **791** |  |  |
| Systolic BP^d^ | 137.9 (25.8) | 137.5 (25.6) | 132.1 (21.6) | 140.5 (27.0) | <0.01 | 139.1 (27.4) | 137.8 (27.5) | 0.02 | 0.04 |
| Oxygen saturation | 96.4 (4.9) | 96.4 (4.8) | 96.7 (5.4) | 96.3 (4.7) | 0.08 | 96.2 (5.2) | 96.2 (4.0) | 0.05 | 0.10 |
| Pulse | 103.5 (23.9) | 104.5 (24.3) | 105.2 (24.3) | 101.9 (23.3) | <0.01 | 103.6 (24.6) | 107.2 (25.0) | 0.85 | <0.01 |
| GCS^d^ category |  |  |  |  | 0.08 |  |  | <0.01 | <0.01 |
| 3-8 | 676 (10.4%) | 256 (9.9%) | 101 (9.7%) | 319 (11.2%) |  | 1219 (19.4%) | 158 (22.2%) |  |  |
| 9-12 | 872 (13.4%) | 360 (13.9%) | 118 (11.3%) | 394 (13.8%) |  | 1021 (16.3%) | 142 (19.9%) |  |  |
| 12-15 | 4944 (76.2%) | 1978 (76.3%) | 821 (78.9%) | 2145 (75.1%) |  | 4034 (64.3%) | 413 (57.9%) |  |  |

^a^ among low acuity

^b^ low acuity vs. high acuity

^c^ among low acuity, high acuity, NOS

^d^ BP indicates blood pressure; GCS indicates Glasgow Coma Scale
