# Supplementary material for: Dispatch Decisions and Emergency Medical Services Response in the Prehospital Care of Status Epilepticus
Source: West J Emerg Med. 2025 May 18;26(3):549–55. doi: 10.5811/westjem.21266 (PMC12208027; doi:10.5811/westjem.21266)
Supplement: Supplementary file 4 [file wjem-26-549-s004.docx]

**Table 4 (Appendix).** Comparison of encounters with a seizure-related EMD code versus non-seizure EMD code with respect to response times, priority level and service level.

|  | EMD is Seizure Related | |
| --- | --- | --- |
|  | No  (n = 4,686) | Yes  (n = 13,829) |
| **Response time (minutes), median (IQR)** | 8.1 (5.8, 11.3) | 8.6 (6.4, 11.6) |
| **Priority, N (%)** |  |  |
| Not emergency | 435 (9.3) | 122 (9.0) |
| Emergency | 4,220 (90.7) | 12,426 (91.0) |
| **Service Level of EMS Unit, N (%)** |  |  |
| BLS | 311 (7.0) | 787 (5.8) |
| ALS + Specialty Critical Care | 4,126 (92.9) | 12,720 (94.1) |
