# Supplementary material for: Dispatch Decisions and Emergency Medical Services Response in the Prehospital Care of Status Epilepticus
Source: West J Emerg Med. 2025 May 18;26(3):549–55. doi: 10.5811/westjem.21266 (PMC12208027; doi:10.5811/westjem.21266)
Supplement: Supplementary file 5 [file wjem-26-549-s005.docx]

**Table 5 (Appendix)**. Distribution of emergency medical dispatch code acuity across priority and service levels, restricting to first responding unit

|  | NOS | Low acuity | High acuity |
| --- | --- | --- | --- |
| **Priority level** |  |  |  |
| Non-emergent | 51 (14.4%) | 445 (17.1%) | 22 (1.0%) |
| Emergent | 298 (84.2%) | 2092 (80.4%) | 2232 (98.9%) |
| **Service level of EMS unit** |  |  |  |
| BLS unit | 12 (3.4%) | 221 (8.5%) | 245 (10.9%) |
| ALS unit | 342 (96.6%) | 2381 (91.5%) | 2012 (89.1%) |
