# Supplementary material for: Dispatch Decisions and Emergency Medical Services Response in the Prehospital Care of Status Epilepticus
Source: West J Emerg Med. 2025 May 18;26(3):549–55. doi: 10.5811/westjem.21266 (PMC12208027; doi:10.5811/westjem.21266)
Supplement: Supplementary file 6 [file wjem-26-549-s006.docx]

**Table 6 (Appendix)** Unadjusted response time^*^ and adjusted**^†^** response times of prehospital encounters for status epilepticus, restricting to first responding unit

|  | Response time (minutes) | | | Adjusted difference in response time (minutes) | |
| --- | --- | --- | --- | --- | --- |
|  | 25th percentile | Median | 75th percentile | Coefficient | 95% Cis |
| **EMD Code** |  |  |  |  |  |
| 12A | 6.2 | 8.4 | 11.6 | Ref |  |
| 12B | 6.2 | 8.2 | 10.9 | -0.9 | -1.3, -0.5 |
| 12C | 6.5 | 8.5 | 11.2 | -0.5 | -0.8, -0.2 |
| 12D | 5.7 | 7.5 | 9.8 | -1.5 | -1.8, -1.2 |
| 12-NOS | 4.8 | 6.6 | 10.2 | -2.2 | -3.1, -1.3 |
| **Priority** |  |  |  |  |  |
| Not emergency | 7.1 | 10.0 | 13.5 | Ref |  |
| Emergency | 5.8 | 7.7 | 10.2 | -1.7 | -2.1, -1.4 |
| **Service Level of EMS Unit** |  |  |  |  |  |
| BLS | 6.6 | 8.2 | 9.9 | Ref |  |
| ALS + Specialty Critical Care | 5.9 | 7.8 | 10.6 | -0.3 | -1.2, 0.5 |

* Defined as the number of minutes between dispatch receiving an emergency call and the ambulance arriving on scene

**†** Adjusted differences in response times were derived by fitting multi-level mixed effects linear regression models with agency as a random effect, to estimate the difference in response time between acuity, priority, and service levels, adjusting for patient age and sex.
