# Supplementary material for: Dispatch Decisions and Emergency Medical Services Response in the Prehospital Care of Status Epilepticus
Source: West J Emerg Med. 2025 May 18;26(3):549–55. doi: 10.5811/westjem.21266 (PMC12208027; doi:10.5811/westjem.21266)
Supplement: Supplementary file 7 [file wjem-26-549-s007.docx]

**Table 7 (Appendix).** Unadjusted response time^*^ and adjusted**^†^** response times of prehospital encounters for status epilepticus, restricting to encounters with receipt of a benzodiazepine.

|  | Response time (minutes) | | | Adjusted difference in response time (minutes) | |
| --- | --- | --- | --- | --- | --- |
|  | 25th percentile | Median | 75th percentile | Coefficient | 95% Cis |
| **EMD Code** |  |  |  |  |  |
| 12A | 6.4 | 8.8 | 12.3 | Ref |  |
| 12B | 7.1 | 9.2 | 12.5 | -0.6 | -1.5, 0.3 |
| 12C | 7.2 | 9.6 | 12.8 | -0.5 | -1.1, 0.1 |
| 12D | 6.2 | 8.3 | 11.1 | -1.9 | -2.4, -1.3 |
| 12-NOS | 5.6 | 7.7 | 10.6 | -2.4 | -3.8, -1.1 |
| **Priority** |  |  |  |  |  |
| Not emergency | 7.7 | 10.7 | 13.8 | Ref |  |
| Emergency | 6.2 | 8.5 | 11.4 | -1.9 | -2.6, -1.1 |
| **Service Level of EMS Unit** |  |  |  |  |  |
| BLS | 7.1 | 7.9 | 9.2 | Ref |  |
| ALS + Specialty Critical Care | 6.3 | 8.6 | 11.6 | 1.8 | -0.4, 3.9 |
|  |  |  |  |  |  |

* Defined as the number of minutes between dispatch receiving an emergency call and the ambulance arriving on scene

**†** Adjusted differences in response times were derived by fitting multi-level mixed effects linear regression models with agency as a random effect, to estimate the difference in response time between acuity, priority, and service levels, adjusting for patient age and sex.
